# Similarity evaluation of DNA sequences based on frequent patterns and entropy

**DOI:** 10.1186/1471-2164-16-S3-S5

**Published:** 2015-01-29

**Authors:** Xiaojing Xie, Jihong Guan, Shuigeng Zhou

**Affiliations:** 1 Shanghai Key Lab of Intelligent Information Processing, and School of Computer Science, Fudan University, 220 Handan Road, 200433 Shanghai, China; 2 Department of Computer Science and Technology, Tongji University, 4800 Cao'an Highway, 201804 Shanghai, China

**Keywords:** DNA sequence comparison, Similarity analysis, Frequent pattern mining, Entropy

## Abstract

**Background:**

DNA sequence analysis is an important research topic in bioinformatics. Evaluating the similarity between sequences, which is crucial for sequence analysis, has attracted much research effort in the last two decades, and a dozen of algorithms and tools have been developed. These methods are based on alignment, word frequency and geometric representation respectively, each of which has its advantage and disadvantage.

**Results:**

In this paper, for effectively computing the similarity between DNA sequences, we introduce a novel method based on frequency patterns and entropy to construct representative vectors of DNA sequences. Experiments are conducted to evaluate the proposed method, which is compared with two recently-developed alignment-free methods and the BLASTN tool. When testing on the *β*-globin genes of 11 species and using the results from MEGA as the baseline, our method achieves higher correlation coefficients than the two alignment-free methods and the BLASTN tool.

**Conclusions:**

Our method is not only able to capture fine-granularity information (location and ordering) of DNA sequences via sequence blocking, but also insensitive to noise and sequence rearrangement due to considering only the maximal frequent patterns. It outperforms major existing methods or tools.

## Background

The rapid development of DNA sequencing technologies has led to a huge number of DNA sequences. It is possible now to obtain large amounts of individual genomes sequenced in one week with less than US$10,000 [1], using the high-throughput sequencing technologies, such as single-molecule sequencing [2] and next-generation sequencing (NGS) [3]. Consequently, DNA sequence analysis faces serious computational challenges due to the huge amounts of data.

Similarity evaluation between sequences is a crucial starting point for analyzing genomic sequences and has a wide range of applications. One important application is to discover the evolutionary relationship between species. This is based on the assumption that two species having similar sequences are close in evolutionary relationship. Another popular application is to search similar sequences in databases. The databases may be huge in size, hence an effective and efficient method for defining and computing the similarity between sequences is badly in need. In addition, there are many reference-based tools and algorithms for sequence compression, such as GReEn [4], RLZ [5] and so on. For these algorithms, the choice of reference sequence has a significant impact on both compression ratio and compression time. Therefore, a preprocessing step that assesses the similarity between sequences and then selects the most similar one with the others as the reference is critical to compression performance.

Due to the importance of sequence similarity analysis, a dozen of algorithms have already been developed. These algorithms can be roughly classed into two categories. The first category is based on sequence alignment, which has been reviewed in [6]. Sequence alignment is powerful for comparison between two related genomes; BLAST [7], FASTA [8] and MEGA [9] are typical sequence alignment tools. However, sequence alignment depends on the orderings of the nucleotides and may be computationally prohibitive. And for comparing long sequences, alignment-based methods take too much time. For example, CUDAlign 3.0[10], one of the state-of-the-art parallel alignment methods, spends more than 5,000 seconds when comparing two sequences of size ~50 MB using 16 GPUs. Fortunately, alignment is not must for similarity analysis.

The second category is alignment-free methods, some of which are based on word (*k*-mer) frequency. The frequencies of words within a DNA sequence are calculated and compared between DNA sequences using statistical distances. A review on these algorithms can refer to [11]. Blaisdell [12] introduced the first word frequency based method and used the Euclidean distance for assessing sequence similarity. Wu *et al*. [13] proposed methods based on Kullback-Leibler discrepancy between frequencies of words, Mahalanobis distances and standardized Euclidean distances under Markov chain models of base composition.

Some other alignment-free methods use geometric representations for DNA sequences. With these methods, sequences are transformed to 2D [14], 3D [15], 4D [16] and 5D [17] spaces and so on. Such graphical representation techniques provide a way to visually measure similarity and dissimilarity between DNA sequences. However, they are time-consuming when comparing long sequences. Recently, there are also methods based on entropy. Li *et al*. [18] introduced the weighted pseudo-entropy, which is used for constructing representative vectors of DNA sequences. And Zhang *et al*. [19] converted DNA sequences into time sequences and then used the approximate entropy [20].

In this paper, we develop a novel method based on **Fr**equent sequential **Pa**tterns and **En**tropy (**FPE **in short) to represent DNA sequences. Concretely, each sequence is first divided into blocks of the same length. Then, a modified PrefixSpan [21] algorithm is used to discover the maximal frequent patterns in each block. Finally, with the probabilities of these patterns, the entropy of each block is calculated. The resulting entropies of the blocks constitute the components of the vector of the sequence. Note that sequences are usually different in size, hence their vectors may have different dimensions.

We conduct extensive experiments to evaluate the proposed method on the *β*-globin genes of 11 species. By evaluating the correlation coefficient between the calculated similarities and the results from MEGA [9], the proposed method FPE achieves higher correlation coefficients than two recently-developed alignment-free methods [14,18] and BLASTN [7]. Further comparison analysis also shows that FPE is more accurate than the two recently-developed methods, and our results agree well with the evolutionary fact.

## Methods

**Figure 1:**
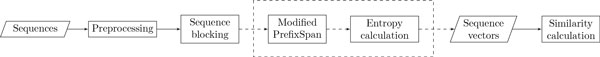
**The pipeline of our method**. The parallelograms stand for input/output modules, and the rectangles indicate functional modules. The two modules enclosed in dashed rectangle run for each block.

The pipeline of our method FPE is shown in Figure 1, which consists of five steps, each of which is implemented by a module. First, each sequence is preprocessed by the *preprocessing *module. Second, each sequence is divided into blocks of the same size by the *sequence blocking *module. Then, a modified PrefixSpan [21] algorithm is used to discover the maximal frequent patterns in each block, which is done by the *modified PrefixSpan *module. The entropy of each block is calculated with the probabilities of discovered patterns in the block, which is finished by the *entropy calculation *module. For each sequence, the entropy values of all blocks form the dimensional components of its final representation vector. Finally, the *similarity calculation *module computes the similarity between any two sequences using their vectors obtained above. We describe each step in detail in the following subsections.

### Preprocessing

For each sequence or genome to be processed, before partitioning it into blocks, a preprocessing is performed, which includes: 1) converting all characters to uppercases; 2) discarding all non-base characters; 3) ignoring all line-breaks in each sequence file.

### Sequence blocking

In our approach, each sequence is first split into several blocks, each of which consists of the same number of consecutive bases. The blocks are independent of each other and the block size can be changed in practice. Note that if the size of the last block is smaller than the specified block size, this block will be discarded. To be clearer, following is an example. In this example, there are two sequences. Assuming that the block size is 20, these two sequences are divided into 3 and 4 blocks, respectively. And the last blocks of size 4 are discarded.

**Figure 2:**
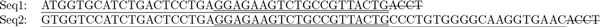
**Two sequences**.

**Example 1 ***Consider the two sequences in *Figure 2*. The block size is 20 and each block is underlined or double underlined. And the last blocks with strikethrough lines are discarded*.

Sequence blocking is an important step that brings two major advantages. First, the blocking strategy can capture fine-granularity information of sequences, including location and ordering information. Second, even for the long sequences, blocking can reduce memory and time consumption for sequence processing.

### Mining maximal frequent patterns from sequences

Most of the previously proposed methods based on word frequency select some fixed-length words and then calculate their frequencies, such as [12] and [13]. As DNA sequences are strings generated from the alphabet {A, G, C, T}, there are totally 4*^k ^*words of length *k*, which are also called *k*-mers in the literature. To describe a sequence well, the parameter *k *should be carefully selected, which possibly depends on the application domain. To avoid information loss, as many as possible patterns (or *k*-mers) should be considered. However, this will unavoidably introduce many low-information patterns (or noise). Keeping this in mind, our method in this paper tries to avoid manually determining the value of parameter *k *while taking all important patterns into account.

In addition, considering that subsequence rearrangements are normal during the biological evolution process, if there are too many rearrangements in the sequences, the results of alignment based methods may be unreliable.

Therefore, in this paper we adopt a modified PrefixSpan [21] algorithm to discover the frequent patterns in DNA sequences and consider only the maximal frequent patterns. This makes our method be considerably tolerant of subsequence rearrangement and noise. In the *Experiments and Results *section, we will present the experimental results that show the proposed method's tolerance of noise and subsequence rearrangements.

PrefixSpan [21] is an efficient algorithm for sequential pattern mining. However, our problem is a little different from traditional sequential pattern mining. Instead of mining a sequence database, we process a single sequence. And there is no gap between the items (subpatterns) in each pattern. We give a formal definition of our problem as follows:

Definition 1 (Mining maximal frequent patterns from a DNA sequence). *Given a DNA sequence S that is a sequence of bases denoted by S = <s*_1_*s*_2 _...*s_n_> where n = *|*S*| *is the length of sequence and s_i_*(1 ≤ *i *≤ *n*) *is a character from the charset *Ω = {*A, T, C, G*}, *and a predefined minimum support threshold s_min_, the support (denoted by sup) of a subsequence of S is the occurrences of the subsequence in S. A subsequence (or pattern) < s_k _s_k+1_*...*s_m _>*(1 ≤ *k *≤ *m *≤ *n*) *is a frequent pattern if its support sup is no less than s_min_. A maximal frequent pattern is the one that none of its super-sequences are frequent. Our problem is to find all the maximal frequent patterns in the DNA sequence*.

In frequent pattern mining, a close concept to *maximal frequent pattern *is *closed frequent pattern*. A *closed frequent pattern *is the one that none of its proper super-sequences have the same support as itself. So maximal frequent patterns must be closed frequent patterns. Actually, mining maximal frequent patterns is done by mining closed frequent patterns.

**Example 2 ***Considering sequence 1 in Example 1. Let s_min _= 2 and check the sub-sequence *〈*CT GA*〉 *in the first block of sequence 1, its sup is 2, so it is a frequent pattern in the first block of sequence 1. Furthermore, there is no any super-sequence of *〈*CT GA*〉 *has a sup that is *≥ *2, so *〈*CT GA*〉 *is a maximal frequent pattern in the first block of sequence 1*.

Before describing the modified PrefixSpan algorithm, we give the definitions of *prefix*, *suffix *and *projected database *as Definition 2 and Definition 3. These definitions are a little different from those in [21]. Note that here we also perform pseudo-projection, instead of physical projection. That is, the projected database contains only the indexes of the suffixes, not the real suffixes. This technique is widely used in the area of frequent pattern mining. In the following algorithms and examples, we just use pseudo-projection for the *α*-*projected database*, *S*|*_α_*.

**Definition 2 (Prefix and Suffix) ***Given a sequence S = <s*_1_*s*_2 _...*s_n_>, we say sequence δ = <s*_1_*s*_2 _...*s_m_> with m < n is a prefix of S, and sequence γ = <s_m+1_s_m+2_*...*s_n_> is the suffix of S with regard to prefix δ, which is denoted as γ = S/δ*.

**Definition 3 (Projected database) ***Let α be a sequential pattern in a sequence S, the α-projected database, denoted as S*|*α, is the collection of suffixes of S with regard to prefix α*.

Algorithm 1 outlines the mining process. Assuming that the current pattern is frequent, the algorithm extends it by appending one base at a time (Line 3), and constructs the corresponding projected database (Line 4). If the extended pattern is frequent and closed (Algorithm 2), then the algorithm recursively calls itself with the extended pattern (Line 8). Therefore, the current pattern is always closed. If the current pattern cannot extend to any frequent pattern (Line 9), it is maximal according to Definition 1. And if the pattern is long enough, it will be saved with its projected database (Line 10).

To check whether a frequent pattern is closed, we adopt the method proposed in [22], which is outlined in Algorithm 2. First, we calculate the start positions of the pattern's occurrences in the input sequence (block), using its pseudo-projected database. That is, we subtract the length of the pattern from each value in the pseudo-projected database (Line 2-3). Then, we check whether the set of these positions is a subset of any single-item-projected database (Line 4). Here, a single item means any base in {A, G, C, T}. If the answer is "yes", the pattern is impossible to be a closed frequent pattern.

**Algorithm 1: **freqPattens(Ω, *s_min_*, *l_min_*, *α*, *S*|*_α_*, *R*) -- the modified PrefixSpan algorithm for mining maximal frequent patterns from DNA sequence.

**Input **: Ω - the charset of bases, namely, {*A, T, C, G*};

*s_min _*- the minimum support threshold;

*l_min _*- the minimum pattern length;

*α *- the current pattern;

*S*|*_α _*- the *α*-projected database;

**Output**: *R *- the set containing all the maximal frequent patterns and their corresponding projected databases;

**1 **isMaximal = true;

**2 foreach *s *∈ Ω do**

**3 **    Append *s *to *α *to form a new pattern *β*;

**4 **    Construct the *β*-projected database *S*|*_β _*;

**5     if |*S*|*_β _*| ≥ *s_min _*then **   /* *β *is frequent */

**6 **        isMaximal = false;

**7         if isClosed(*β*, *S*|*_β_*) then**

**8 **            Call freqPattens(**Ω, *s_min_*, *l_min_*, *β*, *S*|*_β_*, *R***);

**9 if isMaximal = true and |*α*| ≥ *l_min _*then **   /* *α *is maximal */

**10 **    *R *= *R *∪ {*α*, *S*|*_α_*};

**11 return ***R*;

To have a better understanding of the mining process, we give an example as follows:

**Example 3 ***The mining process of the first block of sequence 1 is presented in *Table 1*. Assume that both the minimum support threshold and the minimum pattern length to be 2. Therefore*, 〈*A*〉, 〈*G*〉, 〈*C*〉 *and *〈*T *〉 *are dropped for not reaching the required length. And the patterns whose supports are smaller than 2 are given up, such as *〈*CA*〉 *and *〈*CC*〉 *etc. Patterns that are not maximal, such as *〈*CT *〉 *and *〈*CT G*〉 *etc. are discarded. Note that *〈*GA*〉 *and *〈*T GA*〉 *are still dropped because they are not closed frequent patterns. Take *〈*GA*〉 *for example. First, we compute the start locations of the pattern's occurrences in the sequence block, and obtain the set *{*13 - 2, 20 - 2*} *= *{*11, 18*}*. Obviously, this set is the subset of *〈*T *〉 *-projected database, so *〈*GA*〉 *is not a closed frequent pattern. Then, we obtain three maximal frequent patterns: *〈*AT *〉, 〈*CT GA*〉 *and *〈*T C*〉.

**Table 1:** Illustration of the mining process of the modified PrefixSpan algorithm.

current pattern	extended patterns
〈*C*〉: 7, 10, 14, 16, 17;	〈*CA*〉: 8; 〈*CC*〉: 17; 〈*CG*〉: *Empty*; 〈*CT *〉: 11, 15, 18;

〈*CT *〉: 11, 15, 18;	〈*CT A*〉: *Empty*; 〈*CT C*〉: 16; 〈*CT G*〉: 12, 19; 〈*CT T *〉: *Empty*;

〈*CT G*〉: 12, 19;	〈**CTGA**〉: **13**, **20**; 〈*CT GC*〉: *Empty*; 〈*CT GG*〉: *Empty*; 〈*CT GT *〉: *Empty*;

〈**CTGA**〉: **13**, **20**;	〈*CT GAA*〉: *Empty*; 〈*CT GAC*〉: 14; 〈*CT GAG*〉: *Empty*; 〈*CT GAT *〉: *Empty*;

〈*A*〉: 1, 8, 13, 20;	〈*AA*〉: *Empty*; 〈*AC*〉: 14; 〈*AG*〉: *Empty*; 〈**AT**〉: **2**, **9**;

〈**AT**〉: **2**, **9**;	〈*AT A*〉: *Empty*; 〈*AT C*〉: 10; 〈*AT G*〉: 3; 〈*AT T *〉: *Empty*;

〈*G*〉: 3, 4, 6, 12, 19;	〈*GA*〉: 13, 20; 〈*GC*〉: 7; 〈*GG*〉: 4; 〈*GT *〉: 5;

〈*T *〉: 2, 5, 9, 11, 15, 18;	〈*T A*〉: *Empty*; 〈**TC**〉: **10**, **16**; 〈*T G*〉: 3, 6, 12, 19; 〈*T T *〉: *Empty*;

〈**TC**〉: **10**, **16**;	〈*T CA*〉: *Empty*; 〈*T CC*〉: 17; 〈*T CG*〉: *Empty*; 〈*T CT *〉: 11;

〈*T G*〉: 3, 6, 12, 19;	〈*T GA*〉: 13, 20; 〈*T GC*〉: 7; 〈*T GG*〉: 4; 〈*T GT *〉: *Empty*;

Each row represents one recursive step. The numbers after each pattern represent the starting locations of the suffixes, which are the so-called pseudo-projections. Patterns in bold are maximal.

**Algorithm 2: **isClosed(*α*, *S*|*_α_*) -- determining whether a pattern is closed.

**Input **: *α *- the pattern;

*S*|*_α _*- the *α*-projected database;

**Output**: True - if the pattern is closed;

False - if the pattern is not closed;

**1 ***P *= ∅;    /* initialize the position set */

**2 foreach ***c ***∈ ***S***|***_α _* **do**

**3 **   *P *= *P *∪ {*c *− |*α*|};

**4 if P **⊆*S***|***_A _***or P **⊆*S***|***C ***or P **⊆*S***|***G ***or P **⊆*S***|***T ***then**

**5    return **False;

6 else

**7    return **True;

### Entropy calculation

So far, we have obtained all the maximal frequent patterns for each sequence block. Before evaluating the entropy of each block, the probability of each pattern in the block is computed as follows:

(1) $$p_{pat}=\frac{s_{pat}}{l_{block}-l_{pat}+1}$$

where *s_pat _*is the support of the pattern, *l_block _*is the length of the block, and *l_pat _*is the length of the pattern. It is obvious that the probability of each pattern is positively correlated with its support and its length. When the length of pattern increases to the length of the block, its support will become 1 so that the probability equals 1. And the probability equals 0 when the support drops to 0. As we consider only maximal frequent patterns, and *s_min _*is usually *>*1, those two extreme cases will not happen.

Then, the entropy of a block is defined as below:

(2) $$H=-\frac{1}{\underset{pat\in R}{\sum }s_{pat}}\underset{pat\in R}{\sum }s_{pat}p_{pat}\text{ln}\left(p_{pat}\right)$$

where *R *is the set of maximal frequent patterns mined from the block. Finally, the entropies of all blocks constitute the dimensional components of the final vector of the sequence.

**Example 4 ***From the 1st block of sequence 1 in Example 1, we have obtained three maximal frequent patterns and their probabilities are shown in *Table 2*. Then, it is easy to get its entropy 0.241910 via Eq*. (2).

**Table 2:** Probabilities of patterns.

pattern	*s_pat_*	*l_pat_*	*p_pat_*
〈*AT *〉	2	2	2/(20 − 2 + 1) = 0.105263

〈*CT GA*〉	2	4	2/(20 − 4 + 1) = 0.117647

〈*T C*〉	2	2	2/(20 − 2 + 1) = 0.105263

### Similarity calculation

In the above subsection, we have obtained a representative vector for each sequence.

However, as the vectors may differ in size, there should be a special way to measure

the similarity of two vectors of different sizes.

Let $V_{1}=\left\{V_{1}^{1},V_{1}^{2},\cdots \;,V_{1}^{m}\right\}$ and $V_{2}=\left\{V_{2}^{1},V_{2}^{2},\cdots \;,V_{2}^{n}\right\}$ be the vectors of two different sequences, and assume that 1 ≤ *m *≤ *n*. First, we search a start location *k *in ***V*_2 _**-- the longer vector, such that the following equation holds:

(3) $$|V_{2}^{k}-V_{1}^{1}|=\underset{1\leq i\leq n-m+1}{\text{min}}|V_{2}^{i}-V_{1}^{1}|,1\leq k\leq n-m+1.$$

Then, we calculate the distance between ***V*_1 _**and ***V*_2 _**as follows:

(4) $$dist\left(V_{1},V_{2}\right)=\frac{n}{m}\sqrt{\overset{m}{\underset{i=1}{\sum }}{\left(V_{2}^{k+i-1}-V_{1}^{i}\right)}^{2}}.$$

When *n *equals *m*, *dist*(***V*_1_***, **V*****_2_**) is degenerated to Euclidean distance.

**Example 5 ***Considering the two sequences in Example 1, we obtain **V*****_1 _***= *{*0.241910, 0.238768*} *and **V*****_2 _***= *{*0.244296, 0.238768, 0.254436*}*. Then, we get k *= 1 *and the distance between the two sequences is evaluated as follows:*

$$dist\left(V_{1},V_{2}\right)=\frac{3}{2}\sqrt{{\left(0.241910-0.244296\right)}^{2}+0^{2}}=0.003579.$$

## Experiments and results

### Data used in this work

We choose the *β*-globin genes of 11 species, which are widely used for evaluating the performance of sequence similarity analysis methods. The details of these genes are given in Table 3. Because most existing methods do not provide executable tools or source codes, we do not compare with them on other datasets. All the data are available in the GenBank repository [1].

**Table 3:** Details of *β*-Globin genes of 11 species.

**No**.	species	accession number	location	length (nt)
1	Bovine	[GenBank:X00376]	278-1741	1464

2	Chimpanzee	[GenBank:X02345]	4189-5532	1344

3	Gallus	[GenBank:V00409]	465-1810	1346

4	Goat	[GenBank:M15387]	279-1749	1471

5	Gorilla	[GenBank:X61109]	4538-5881	1344

6	Human	[GenBank:U01317]	62187-63610	1424

7	Lemur	[GenBank:M15734]	154-1595	1442

8	Mouse	[GenBank:V00722]	275-1462	1188

9	Opossum	[GenBank:J03643]	467-2488	2022

10	Rabbit	[GenBank:V00882]	277-1419	1143

11	Rat	[GenBank:X06701]	310-1505	1196

The *location *column gives the start/end location of the sequence of each gene.

[1]http://www.ncbi.nlm.nih.gov/genbank

### Experimental setting

We compare our method (FPE) with BLASTN [7] and two recently-developed alignment-free methods [14,18]. The results of the MEGA [9] software are used as the ground truth. The experiments are done on a PC with Intel Xeon E5606 2.13GHz CPU and 8 GB memory. The operating system is Ubuntu 10.04. By default, we run our method with the minimum support threshold being 3 and the minimum pattern length being 2.

### Experimental results and analysis

#### Preservation of fine-granularity information

**Figure 3:**
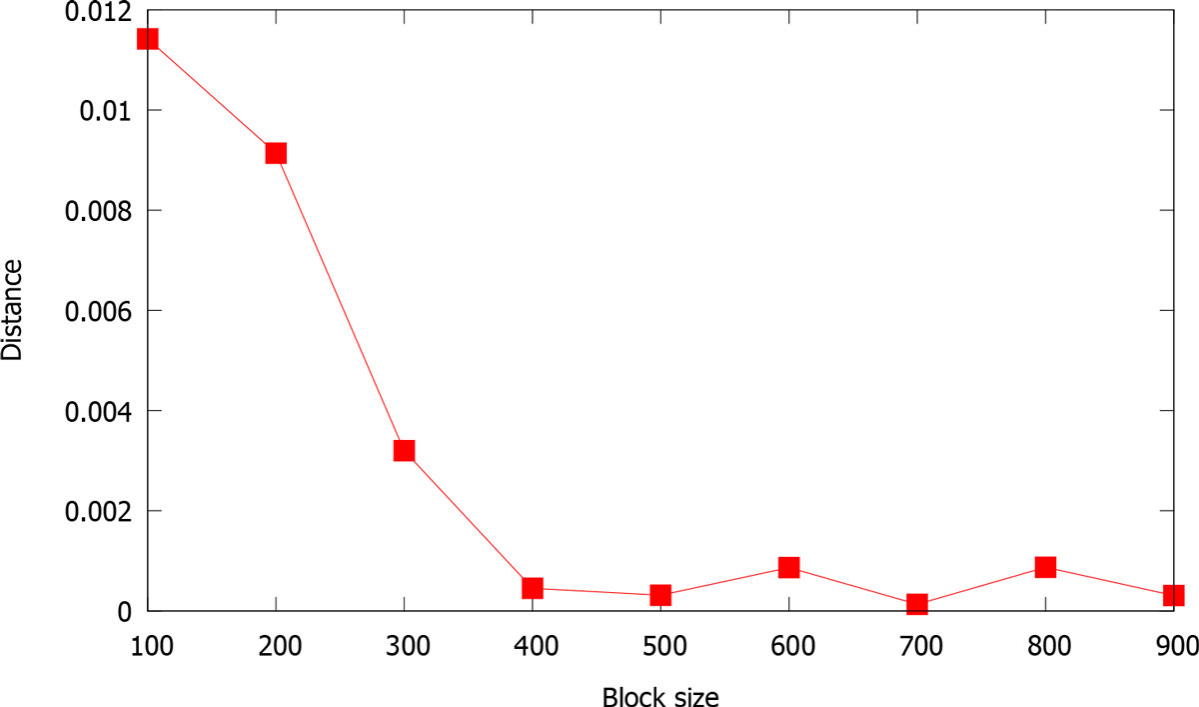
The distance between human and gorilla *vs*. block size.

Our method can capture fine-granularity information of sequences via blocking. To show this, we examine how the distance between human sequence and gorilla sequence changes with block size. The results are presented in Figure 3. It can be seen that as block size decreases, the distance becomes larger, because smaller block size means finer information exploited in the representative vectors. This is a proof that our method can capture fine-granularity information.

#### Tolerance of sequence rearrangement

**Figure 4:**
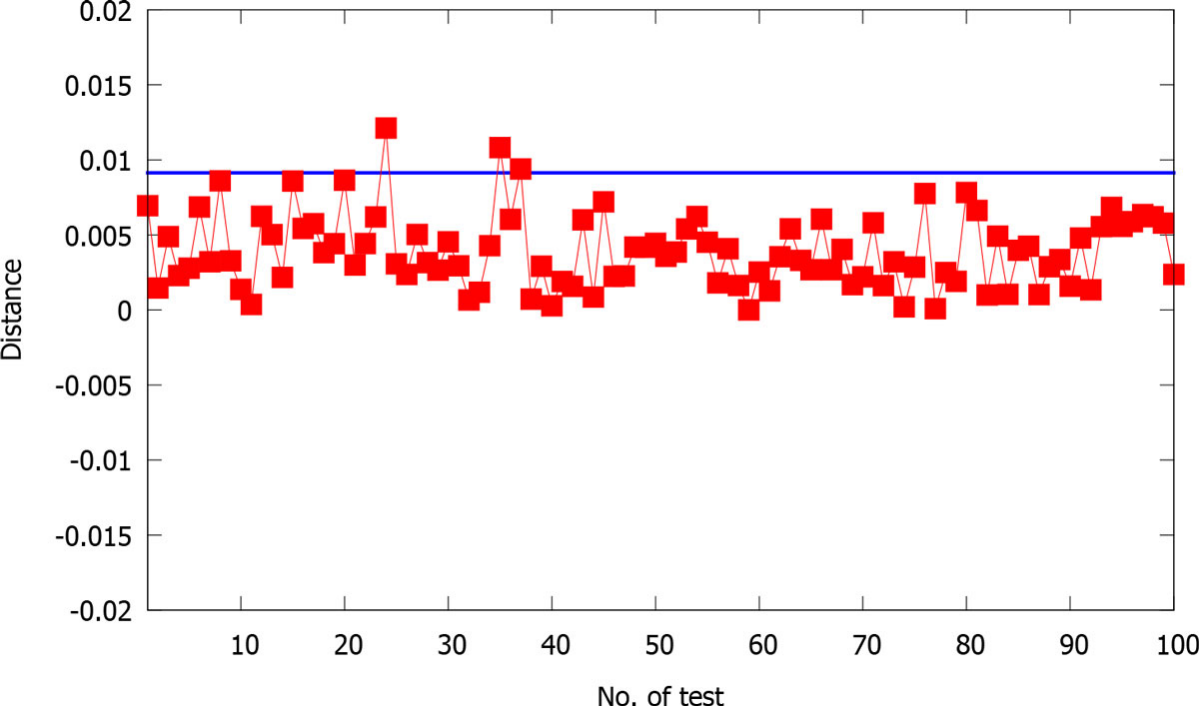
**The distances between original human sequence segments and the corresponding shuffled ones**. The horizontal blue line indicates the distance between sequences of human and gorilla. The horizontal axis indicates the test label from 1 to 100.

Figure 4 shows the distances between original human sequence segments and the corresponding shuffled segments. Here, we set the block size to 200. We repeat the following process 100 times: randomly choose a consecutive segment whose size is 1/10 of the human sequence length, shuffle the bases in the segment, and then compute the distance between the original segment and the shuffled one. After that, we average the distances over the 100 tests. The averaged distance is 0.003946 with the standard deviation being 0.002451. As the sequences of human and gorilla are very similar, we use their distance (0.009138) for comparison, which is also drawn in Figure 4 by the blue line. We can see that the distance between the original human sequence segments and the corresponding shuffled ones is quite small, thus we conclude that our method is tolerant of sequence rearrangement.

#### Tolerance of noise

**Figure 5:**
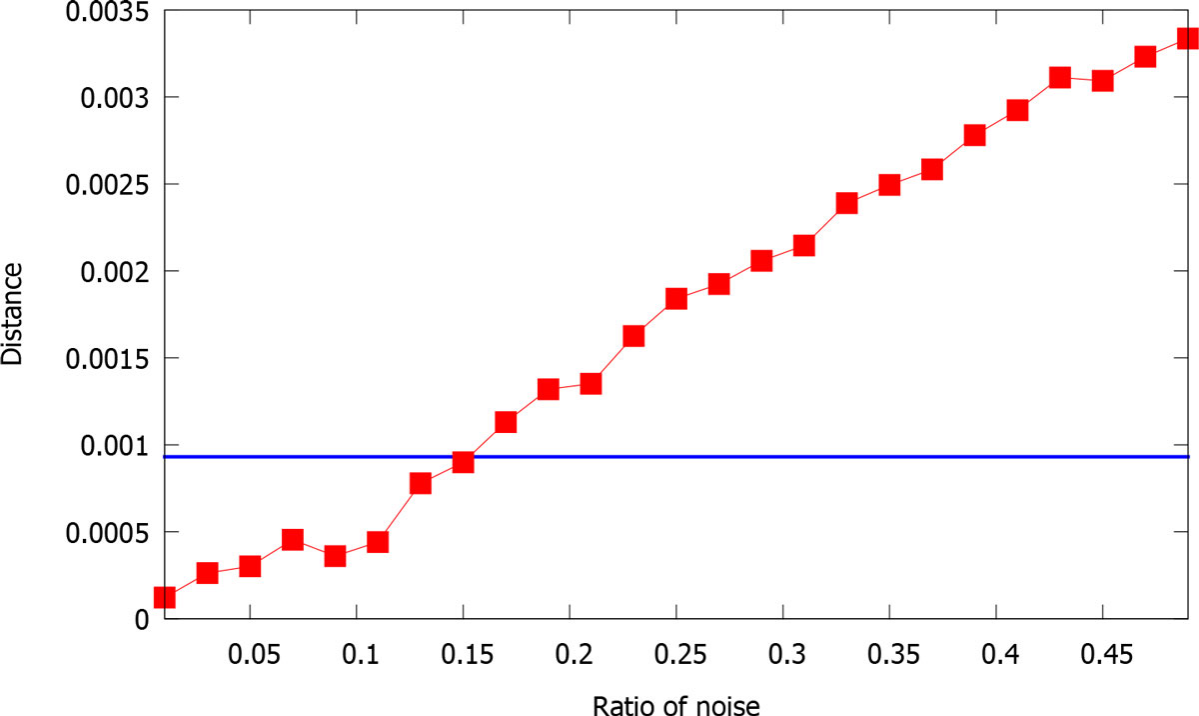
**The averaged distance between the original human sequence and the contaminated sequences when noise ratio increases from 0.01 to 0.5**. The horizontal blue line indicates the distance between the sequences of human and gorilla. The horizontal axis is the ratio of noise added to the sequence.

To test noise tolerance of our method, we randomly insert some letters selected from the charset {*A, T, C, G*} into the human sequence. We define the noise ratio as the proportion of the number of inserted letters over the length of the sequence. Given a noise ratio, we first generate 100 contaminated sequences, then calculate the distance between the original sequence and each of the 100 contaminated sequences, and finally we evaluate the average distance over the 100 tests. Figure 5 shows the averaged distance between the original sequence and the contaminated sequences when noise ratio increases from 0.01 to 0.5. Here, we set the block size to 3000, which is much larger than the length of human sequence and the contaminated ones, so there is actually no blocking over the sequences. We can see that the distance increases as more noise is added. However, when noise ratio is less than 0.15, the distance is smaller than the distance between the human sequence and the gorilla sequence, which is 0.000931 under this setting. This demonstrates that our method is substantially tolerant to noise.

#### Similarity analysis

**Figure 6:**
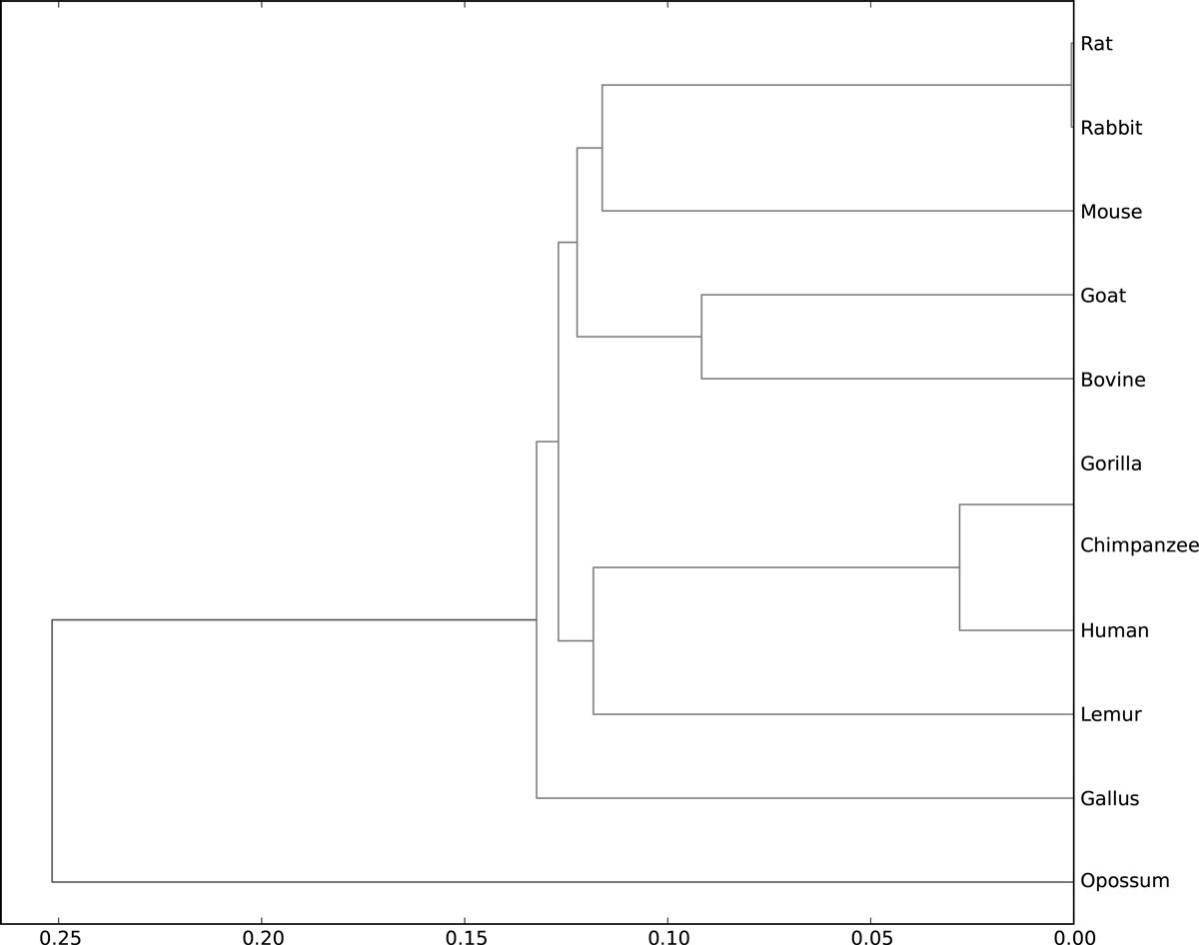
**The dendrogram of the 11 tested species based on the similarity matrix in Table 4**. The dendrogram is generated by using the python SciPy library (http://www.scipy.org/).

Table 4 gives the pairwise distance matrix between the genes of the 11 tested species. Here, we set the minimum pattern length to 8, the minimum support threshold to 2, and the block size to 200. And Figure 6 shows the phylogenetic tree based on this matrix. From Figure 6, we can see that gorilla, chimpanzee and human are close to each other. Lemur is closer to them than the other species. Goat and bovine are in the same group. Rat, rabbit and mouse are also relatively close. And gallus and opossum are quite dissimilar with the other species. This is because gallus is the only non-mammal and opossum is the most remote species among the mammals. The results are similar to those of [14] and [18], and agree well with the evolutionary fact.

**Table 4:** Pairwise distance matrix of *β*-Globin genes of 11 species.

	bovine	chimpanzee	gallus	goat	gorilla	human	lemur	mouse	opossum	rabbit	rat
Bovine	0.0000	0.0782	0.1112	0.0474	0.0782	0.0670	0.0824	0.0666	0.1202	0.0666	0.0663

Chimpanzee	0.0782	0.0000	0.0679	0.0957	0.0000	0.0000	0.0558	0.0568	0.1579	0.0569	0.0569

Gallus	0.1112	0.0679	0.0000	0.1239	0.0679	0.0792	0.0962	0.0806	0.1935	0.0805	0.0805

Goat	0.0474	0.0957	0.1239	0.0000	0.0957	0.0820	0.0675	0.0939	0.0994	0.0939	0.0938

Gorilla	0.0782	0.0000	0.0679	0.0957	0.0000	0.0000	0.0558	0.0568	0.1579	0.0569	0.0569

Human	0.0670	0.0000	0.0792	0.0820	0.0000	0.0000	0.0478	0.0663	0.1537	0.0664	0.0664

Lemur	0.0824	0.0558	0.0962	0.0675	0.0558	0.0478	0.0000	0.0942	0.1428	0.0945	0.0944

Mouse	0.0666	0.0568	0.0806	0.0939	0.0568	0.0663	0.0942	0.0000	0.1643	0.0672	0.0671

Opossum	0.1202	0.1579	0.1935	0.0994	0.1579	0.1537	0.1428	0.1643	0.0000	0.1643	0.1643

Rabbit	0.0666	0.0569	0.0805	0.0939	0.0569	0.0664	0.0945	0.0672	0.1643	0.0000	0.0003

Rat	0.0663	0.0569	0.0805	0.0938	0.0569	0.0664	0.0944	0.0671	0.1643	0.0003	0.0000

#### Comparison with existing methods

We compare our method with three existing methods, including two recently-developed alignment-free methods and BLASTN 2.2.29+ [7]. One of the two compared alignment-free methods was developed by Li *et al*. in 2011 [18], the other was developed by Yu and Huang in 2013 [14]. As in [14], the results obtained by the MEGA [9] software -- a famous alignment-based tool, are used as the baseline. We also calculate the Pearson correlation coefficient between the results of MEGA and those of our method and the three compared methods. Table 5 shows the distances between human and other species via the five different methods (including MEGA). Here, the last column lists the Pearson correlation coefficients between the results of MEGA 5.2 and those of our method and the three compared methods. For BLASTN, we use the maximum scores and normalize them to the range between 0 and 1 as the similarity, then the distance is 1 minus the similarity. As we can see, our method achieves the highest correlation with MEGA. This shows that our method can calculate the similarity between DNA sequences more accurately.

**Table 5:** Comparison of the distances between human and the other tested species.

	Bovine	Chimpanzee	Gallus	Goat	Gorilla	Lemur	Mouse	Opossum	Rabbit	Rat	Correlation coefficient
MEGA 5.2	0.4485	0.0095	0.8456	0.4696	0.0117	0.2423	0.4815	0.8337	0.4083	0.4935	-

BLASTN 2.2.29+[7]	0.8600	0.0896	0.9880	0.8765	0.0896	0.6643	0.9026	1.0000	0.8423	0.9182	0.8912

Method of [14]	22.4257	5.3704	23.5869	26.8209	5.3704	25.2515	25.8007	25.9952	20.5706	27.0102	0.7569

Method of [18]	0.1000	0.0100	0.2150	0.1050	0.0110	0.0550	0.0830	0.0890	0.0700	0.0620	0.8318

FPE	0.0670	0.0000	0.0792	0.0820	0.0000	0.0478	0.0663	0.1537	0.0664	0.0664	**0**.**8966**

Note that the results from [14] are based on the sequence of the first exon in each *β*-globin gene.

**Figure 7:**
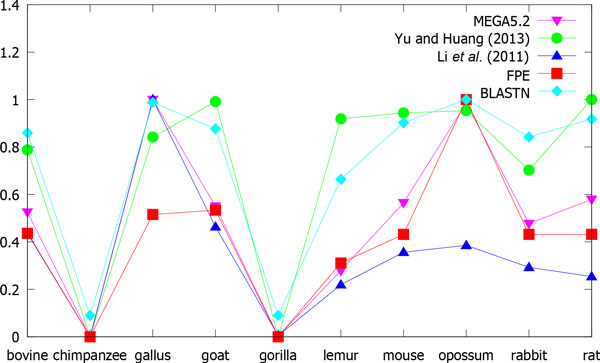
**Comparing the distances evaluated by different methods**. The vertical axis indicates the normalized distances between human and the other tested species, as shown in Table 5.

To make it clearer, we also present the distances between human and the other tested species calculated by our method and the three compared methods as well as MEGA in Figure 7. Here, the distance is normalized to the range between 0 and 1. We can see that the distance between opossum and human evaluated by the method proposed in [18] is not large enough, which violates the fact that opossum is the most remote species among the mammals; while the distance between lemur and human obtained by the method proposed in [14] is too large, which also disagrees with the evolutionary fact. However, for our method, the curve is more similar to that calculated by the MEGA method, which again shows that our method achieves the highest correlation with the ground truth.

## Discussion

Existing methods for measuring similarity/dissimilarity between DNA sequences roughly fall into two types: alignment-based methods and alignment-free methods. As alignment-based methods may be computationally expensive and not scalable to huge datasets, alignment-free methods have been extensively investigated recently.

For the alignment-free methods, on the one hand, algorithms based on word frequency are sensitive to the length of words used, and may include noise if taking all the words into account. On the other hand, algorithms based on geometric representation provide visual comparison among DNA sequences locally and globally. However, for long sequences, these methods may require too much computation overhead and memory. Recently, entropy-based methods provide simple representations for the sequences, but they are prone to lost some important information, for example, location and ordering information. As they treat all symbols or patterns equally, they may also include noise.

Our method is based on frequent patterns and entropy. As we consider only the maximal frequent patterns in a sequence, our method can considerably tolerate noise and sequence rearrangements. Furthermore, by using the blocking strategy, fine-granularity information of the sequences can be captured.

However, for more accurately evaluating the similarity between two sequences, some parameters have to be tuned. In our algorithm, the block size, the minimum support threshold and the minimum pattern length can be changed for different sequences. Note that to compare two sequences, the block size should be the same. And the larger the block size, the more information will be lost. Here, we present the following rule of thumb for parameter tuning:

1) By experiments, we found that it is better to set the block size less than 400, which can be also observed from Figure 3.

2) Once the block size is determined, there may be an optimum pair of the minimum support threshold and the minimum pattern length to be determined.

3) We observed a negative correlation between the minimum support threshold and the minimum pattern length. This means that if the minimum support threshold is large, the minimum pattern length should be set to relatively small. Otherwise, we may get no maximal frequent patterns in some blocks, and thus lose too much information.

Finally, we want to point out that different methods have their own application scenarios. For example, methods based on geometric representation are very powerful tools for visually and intuitively analyzing the sequences. As for our method, we provide an effective way to represent a DNA sequence to a vector, which is suitable for searching similar sequences in databases or acting as a preprocessing step for other applications. Considering that our method has been shown to provide more accurate distances, so it is also suitable for discovering evolutionary relationships.

## Conclusion

This paper presents a novel method based on frequent patterns and entropy to represent the DNA sequences and evaluate their similarities. By using blocking technique, our method can capture fine-granularity information of sequences. Our method can also tolerate noise and sequence rearrangements because we take only the maximal frequent patterns into account. Experiments over the *β*-globin genes of 11 species show that our method achieves more accurate distances than two recently-developed alignment-free methods and the BLASTN tool.

## Competing interests

The authors declare that they have no competing interests.

## Authors' contributions

Shuigeng Zhou and Jihong Guan conceived the study, and revised the manuscript. Xiaojing Xie designed the algorithm, performed all experiments and data analysis, and drafted the manuscript.

## Declarations

This work was supported by China 863 Program (grant No. 2012AA020403) and National Natural Science Foundation of China (NSFC) under grants No. 61173118 and No. 61272380.

This article has been published as part of *BMC Genomics *Volume 16 Supplement 3, 2015: Selected articles from the 10th International Symposium on Bioinformatics Research and Applications (ISBRA-14): Genomics. The full contents of the supplement are available online at http://www.biomedcentral.com/bmcgenomics/supplements/16/S3.

## References

[B1] Loh P-R Baym M Berger B Compressive genomics Nature Biotechnology 30 7 627 630 10.1038/nbt.2241 22781691

[B2] Pushkarev D Neff NF Quake SR Single-molecule sequencing of an individual human genome Nature Biotechnology 2009 27 9 847 850 10.1038/nbt.1561 19668243 PMC4117198

[B3] Horner D Pavesi G Castrignanò T De Meo P Liuni S Sammeth M Picardi E Pesole G Bioinformatics approaches for genomics and post genomics applications of next-generation sequencing Briefings in Bioinformatics 2010 11 2 181 197 10.1093/bib/bbp046 19864250

[B4] Pinho AJ Pratas D Garcia SP Green: a tool for efficient compression of genome resequencing data Nucleic Acids Research 2012 40 4 27 27 10.1093/nar/gkr1124 22139935 PMC3287168

[B5] Kuruppu S Puglisi SJ Zobel J Optimized relative lempel-ziv compression of genomes Proceedings of the Thirty-Fourth Australasian Computer Science Conference 2011 113 Australian Computer Society, Inc 91 98

[B6] Durbin R Biological Sequence Analysis: Probabilistic Models of Proteins and Nucleic Acids 1998 Cambridge University Press, UK

[B7] Altschul SF Madden TL Sch¨affer AA Zhang J Zhang Z Miller W Lipman DJ Gapped blast and psi-blast: a new generation of protein database search programs Nucleic Acids Research 1997 25 17 3389 3402 10.1093/nar/25.17.3389 9254694 PMC146917

[B8] Pearson WR Rapid and sensitive sequence comparison with fastp and fasta Methods in Enzymology 1990 183 63 98 2156132 10.1016/0076-6879(90)83007-v

[B9] Tamura K Peterson D Peterson N Stecher G Nei M Kumar S Mega5: molecular evolutionary genetics analysis using maximum likelihood, evolutionary distance, and maximum parsimony methods Molecular Biology and Evolution 2011 28 10 2731 2739 10.1093/molbev/msr121 21546353 PMC3203626

[B10] Sandes EFdO Miranda G de Melo AC Martorell X Ayguade E Cudalign 3.0: Parallel biological sequence comparison in large gpu clusters Cluster, Cloud and Grid Computing (CCGrid), 2014 14th IEEE/ACM International Symposium On 2014 IEEE 160 169

[B11] Vinga S Almeida J Alignment-free sequence comparison--a review Bioinformatics 2003 19 4 513 523 10.1093/bioinformatics/btg005 12611807

[B12] Blaisdell BE A measure of the similarity of sets of sequences not requiring sequence alignment Proceedings of the National Academy of Sciences 1986 83 14 5155 5159 10.1073/pnas.83.14.5155 PMC323909 3460087

[B13] Wu T-J Hsieh Y-C Li L-A Statistical measures of dna sequence dissimilarity under markov chain models of base composition Biometrics 2001 57 2 441 448 10.1111/j.0006-341X.2001.00441.x 11414568

[B14] Yu H-J Huang D-S Graphical representation for dna sequences via joint diagonalization of matrix pencil IEEE Journal of Biomedical and Health Informatics 2013 17 3 503 511 24592449 10.1109/titb.2012.2227146

[B15] Jafarzadeh N Iranmanesh A C-curve: a novel 3d graphical representation of dna sequence based on codons Mathematical Biosciences 2013 241 2 217 224 10.1016/j.mbs.2012.11.009 23246806

[B16] Chi R Ding K Novel 4d numerical representation of dna sequences Chemical Physics Letters 2005 407 1 63 67

[B17] Liao B Li R Zhu W Xiang X On the similarity of dna primary sequences based on 5-d representation Journal of Mathematical Chemistry 2007 42 1 47 57 10.1007/s10910-006-9091-z

[B18] Li C Ma H Zhou Y Wang X Zheng X Similarity analysis of dna sequences based on the weighted pseudo-entropy Journal of Computational Chemistry 2011 32 4 675 680 10.1002/jcc.21656 20890910

[B19] Zhang X Zhou XA Yu YH Similarity analysis of dna using improved approximate entropy Biomedical Engineering and Biotechnology, International Conference 2012 IEEE 511 514

[B20] Pincus SM Approximate entropy as a measure of system complexity Proceedings of the National Academy of Sciences 1991 88 6 2297 2301 10.1073/pnas.88.6.2297 PMC51218 11607165

[B21] Pei J Han J Mortazavi-Asl B Wang J Pinto H Chen Q Dayal U Hsu M-C Mining sequential patterns by pattern-growth: The prefixspan approach Knowledge and Data Engineering, IEEE Transactions 2004 16 11 1424 1440 10.1109/TKDE.2004.77

[B22] Wang J Han J Bide: Efficient mining of frequent closed sequences Data Engineering, 2004. Proceedings. 20th International Conference 2004 IEEE 79 90

